# Compartmentalized Reconstitution of Post-*s*qualene Pathway for 7-Dehydrocholesterol Overproduction in *Saccharomyces cerevisiae*

**DOI:** 10.3389/fmicb.2021.663973

**Published:** 2021-05-21

**Authors:** Xiao-Jing Guo, Ming-Dong Yao, Wen-Hai Xiao, Ying Wang, Guang-Rong Zhao, Ying-Jin Yuan

**Affiliations:** ^1^Frontier Science Center for Synthetic Biology and Key Laboratory of Systems Bioengineering (Ministry of Education), School of Chemical Engineering and Technology, Tianjin University, Tianjin, China; ^2^Collaborative Innovation Center of Chemical Science and Engineering (Tianjin), Tianjin University, Tianjin, China

**Keywords:** compartmentation, endoplasmic reticulum, lipid bodies, post-squalene pathway, 7-dehydrocholesterol, *Saccharomyces cerevisiae*

## Abstract

7-Dehydrocholesterol (7-DHC) is the direct precursor to manufacture vitamin D_3_. Our previous study has achieved 7-DHC synthesis in *Saccharomyces cerevisiae* based on the endogenous post-squalene pathway. However, the distribution of post-squalene enzymes between the endoplasmic reticulum (ER) and lipid bodies (LD) might raise difficulties for ERG proteins to catalyze and deliver sterol intermediates, resulting in unbalanced metabolic flow and low product yield. Herein, we intended to rearrange the subcellular location of post-squalene enzymes to alleviate metabolic bottleneck and boost 7-DHC production. After identifying the location of DHCR24 (C-24 reductase, the only heterologous protein for 7-DHC biosynthesis) on ER, all the ER-located enzymes were grouped into four modules: ERG1/11/24, ERG25/26/27, ERG2/3, and DHCR24. These modules attempted to be overexpressed either on ER or on LDs. As a result, expression of LD-targeted DHCR24 and ER-located ERG1/11/24 could promote the conversion efficiency among the sterol intermediates to 7-DHC, while locating module ERG2/3 into LDs improved the whole metabolic flux of the post-squalene pathway. Coexpressing LD-targeted ERG2/3 and DHCR24 (generating strain SyBE_Sc01250035) improved 7-DHC production from 187.7 to 308.2 mg/L at shake-flask level. Further expressing ER-targeted module ERG1/11/24 in strain SyBE_Sc01250035 dramatically reduced squalene accumulation from 620.2 mg/L to the lowest level (by 93.8%) as well as improved 7-DHC production to the highest level (to 342.2 mg/L). Then targeting module ERG25/26/27 to LDs further increased 7-DHC titer to 360.6 mg/L, which is the highest shake-flask level production for 7-DHC ever reported. Our study not only proposes and further proves the concept of pathway compartmentalized reconstitution to regulate metabolic flux but also provides a promising chassis to produce other steroidal compounds through the post-squalene pathway.

## Background

**FIGURE 1:**
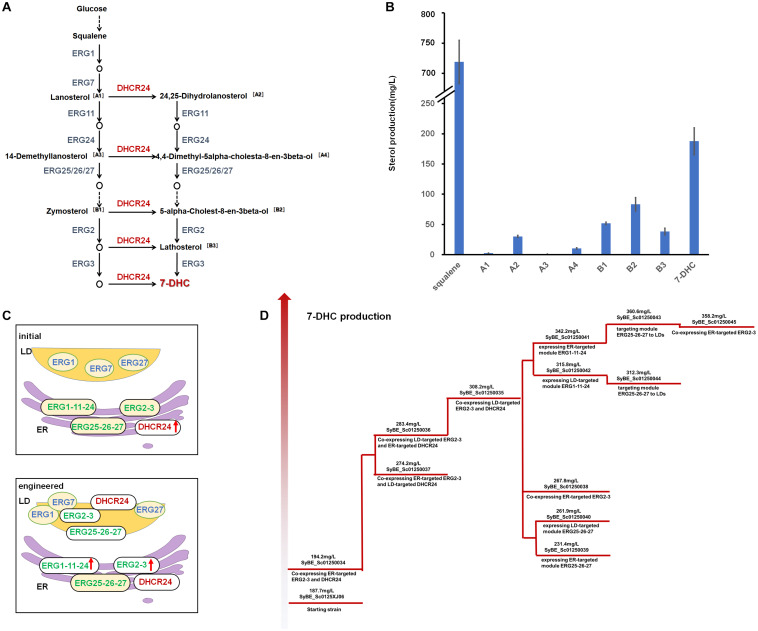
The 7-dehydrocholesterol (7-DHC) biosynthesis pathway and the engineering strategies applied in this study. **(A)** The 7-DHC biosynthesis pathway in yeast. The code for each sterol intermediate is labeled beside the compound. Endogenous genes are in dark gray, and the heterologous gene is in red. **(B)** Metabolites of the post-squalene pathway accumulations in the control strain SyBE_Sc0125XJ06. **(C)** Schematic representation of the engineering strategies to enhance 7-DHC production in *Saccharomyces cerevisiae*. Enzyme distribution of the post-squalene pathway for the strain with the highest 7-DHC production in this study compared with the starting strain. LD-targeted or LD/ER dual localized endogenous enzymes of wild yeast are in blue; ER-targeted endogenous enzymes of wild yeast are in green; heterologous enzyme DHCR24 is in red. Backgrounds of the enzymes without modification are yellow. The up arrows represent protein overexpression. **(D)** Schematic representation of all the strains constructed in this study with their 7-DHC production and applied strategies.

7-Dehydrocholesterol (7-DHC) is a high-valued sterol, which could be converted into vitamin D_3_ (VD_3_) when exposed to UV (Bendik et al., 2014). Besides being an essential nutriment, VD_3_ and its derivatives [such as 25-OH-VD_3_ and 1,25-(OH)_2_-VD_3_] are also medicines for many diseases (Bendik et al., 2014; de Sousa Almeida-Filho et al., 2017; Lu et al., 2017; Ali, 2020; D’Avolio et al., 2020; Kanstrup et al., 2020; Maghbooli et al., 2020). So far, *de novo* biosynthesis of 7-DHC has been realized in *Saccharomyces cerevisiae* by introducing heterologous Δ^24^-dehydrocholesterol reductase (DHCR24) based on endogenous ergosterol synthesis pathway (Lang and Veen, 2011; Hohmann et al., 2012; Su et al., 2015; Guo et al., 2018), in which squalene is a key node separating the pre-squalene pathway and post-squalene pathway. The engineering approaches applied on pre-squalene pathway hardly affect the metabolic flux through the post-squalene pathway. As there is only one heterologous enzyme involved in 7-DHC synthesis (Figure 1A), therefore, the production of 7-DHC largely depends on the behavior of the post-squalene pathway. Although 7-DHC production has been greatly boosted by many metabolic engineering strategies, such as blocking the competitive ergosterol accumulation (Hohmann et al., 2012; Su et al., 2015), improving precursor supply (Lang and Veen, 2011; Su et al., 2015; Guo et al., 2018), screening the key enzyme sources (Hohmann et al., 2012; Guo et al., 2018), alleviating redox imbalance (Su et al., 2015), as well as regulating lipid metabolism (Guo et al., 2018), a large amount of post-squalene intermediates [e.g., squalene, zymosterol (B1), and 5alpha-Cholest-8-en-3beta-ol (B2)] still accumulated within the producing strains, such as our previously constructed strain SyBE_Sc0125XJ06 (Guo et al., 2018; Figure 1B). In this strain, all the mevalonate (MVA) pathway genes (*ERG10*, *ERG13*, *tHMG1*, *ERG12*, *ERG8*, *ERG19*, *IDI1*, and *ERG20*) were overexpressed for enhancing precursor supply; *ERG5* and *ERG6* were knocked out to block the branch pathway. Two copies of heterologous gene *DHCR24* were introduced and overexpressed under GAL1 promoter. Notably, squalene accumulation is the biggest obstacle at the current stage. There was 187.7 mg/L of 7-DHC obtained along with 718.9 mg/L of squalene produced at shake-flask level (Figure 1B). These metabolic bottlenecks within the post-squalene pathway seriously obstruct the conversion from upstream metabolites to 7-DHC.

Balancing the expression level of the modules besides the metabolic node is a promising strategy to reduce the bottleneck of our desired pathway. There are nine ERG proteins catalyzing 16 steps of reactions within the post-squalene pathway (Figure 1A). Our previous study once tempted to tune the expression level of DHCR24 (Guo et al., 2018) and every post-squalene proteins (Zhang et al., 2016). However, the effect of these modification on the metabolic flow of the post-squalene pathway is very limited, which pushed us to seek other solutions for this issue. Fortunately, the subcellular distribution of these ERG proteins gives us a hint of the potential solution to those metabolic bottlenecks. As shown in Figure 1C, post-squalene enzymes are not organized in one single organelle in the *S. cerevisiae* cytoplasm, but distribute between the endoplasmic reticulum (ER) and lipid bodies (LDs). To be specific, ERG1 and ERG27 are found on both organelles, while ERG7 is the only enzyme that located only in LDs (Milla et al., 2002). The rest of the ERG proteins locate in the ER (Ott et al., 2005). This distribution pattern might raise difficulties for ERG proteins to catalyze and deliver sterol intermediates, resulting in unbalanced metabolic flow and even low product yield of the desired product. Meanwhile, excess sterols generated in yeast cells are always stored in LDs in their ester forms (Zweytick et al., 2000; Jacquier and Schneiter, 2012). Ott et al. (2005) once reported the existence of sterol metabolites (e.g., B1) accumulated in LDs at significant levels in the strain with the same Δ*ERG6* background as our strain SyBE_Sc0125XJ06 (Figure 1C). These parts of sterols are hard to be catalyzed by ER-located proteins like ERG2 for its direct substrate B1. Moreover, organelles can provide different intracellular environments for enzyme catalysis, such as different pH environments or redox states. Therefore, a certain organelle may be more conducive to the catalysis of a specific protein. It was reported that mitochondria provided a preferable environment for amorphadiene synthase (ADS) activity, which caused enhanced amorphadiene production when ADS was overexpressed in yeast mitochondria compared with in the cytosol (Yuan and Ching, 2016). Thus, the subcellular distribution of post-squalene enzymes might be the limiting factor for the metabolic flux of this pathway. In addition, compartmentalized reconstitution, which used to be an effective way to improve outputs of many natural products (Lin et al., 2017; Sadre et al., 2019; Yang et al., 2019; Yee et al., 2019; Jia et al., 2020; Liu et al., 2020), also would be a promising solution to boost 7-DHC titer when applied in the post-squalene pathway.

In this study, after identifying the location of the only heterologous enzyme DHCR24 (C-24 reductase) on ER like most of the other ERG enzymes, all the ER-located proteins within the post-squalene pathway were chosen to prove the concept of pathway compartmentalized reconstitution on regulation of metabolic flux. Strain SyBE_Sc0125XJ06 with double deletion of *ERG5/ERG6* and two copies of gallus DHCR24 was employed as the starting strain. The whole post-squalene pathway was divided into two parts by B1, and all the ER-located enzymes were grouped into four modules: ERG1/11/24, ERG25/26/27, ERG2/3, and DHCR24. These modules were individually overexpressed on ER or in LDs (Figure 1D). Also, their effects on the production of 7-DHC as well as the accumulation of sterol intermediates were investigated, revealing an effective effort of pathway compartmentalization to pull down the metabolic flux through the post-squalene pathway. Eventually, targeting modules ERG25/26/27, ERG2/3, and DHCR24 into LDs along with supplementing another copy of ER-located ERG1/11/24 promoted 7-DHC production from the initial 187.7–360.6 mg/L at shake-flask level in strain SyBE_SC01250043, while the squalene accumulation was dramatically reduced by 95.5% to 32.4 mg/L. Although adding another copy of ER-located ERG2/3 into strain SyBE_SC01250043 did not further improve the 7-DHC titer, it increased the 7-DHC ratio to 39% of the total sterols, which is 2.29-fold of the initial ratio. This study achieves the highest shake-flask level titer of 7-DHC as known, which demonstrates a promising strategy to regulate metabolic flux by pathway compartmentalized reconstitution. In addition, sterol intermediates within the post-squalene pathway are also precursors to manufacture other valuable steroidal compounds; therefore, this study also provides an efficient chassis for microbial steroidogenesis.

## Results and Discussion

### Identifying the Subcellular Location of the Desired Protein in *Saccharomyces cerevisiae*

**FIGURE 2:**
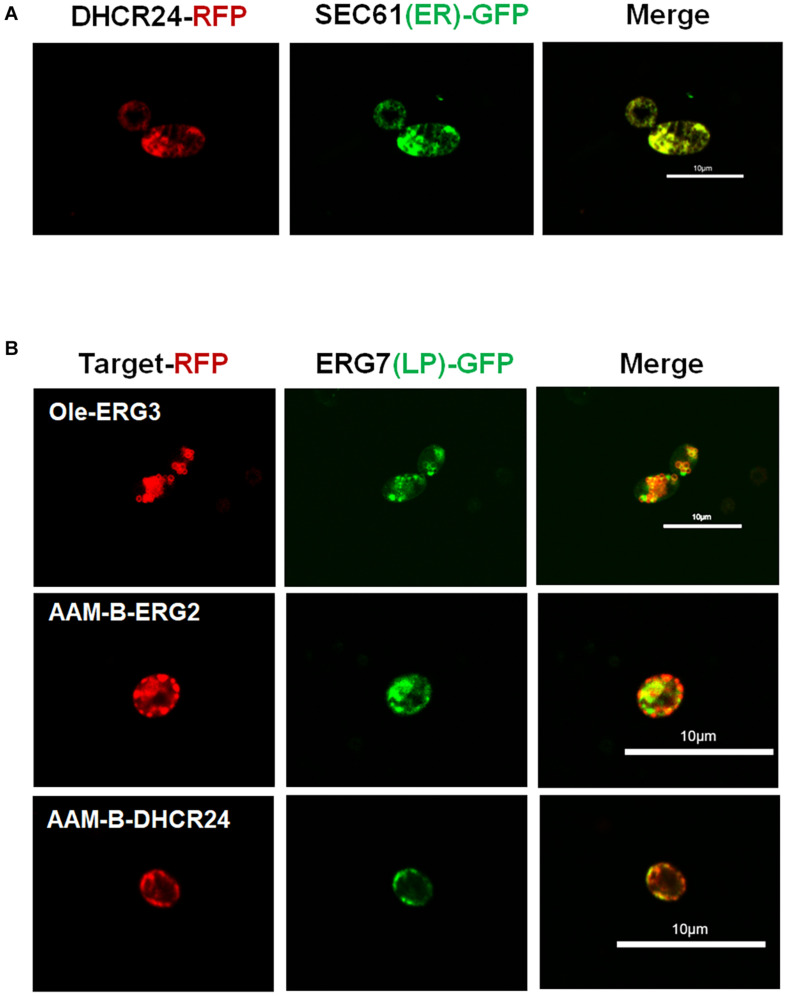
Identifying the subcellular localization of gallus DHCR24 and verifying the reported LD localization sequence in *S. cerevisiae*. **(A)** Analysis of subcellular localization of gallus DHCR24 in *S. cerevisiae*. Sec61 is the specific marker of yeast ER. **(B)** Analysis of subcellular localization of two LD tags including oleosin (Ole) and AAM-B. ERG7 is the specific marker of yeast LDs. **(C)** Measurement of the expression levels of ER-/LD-located protein. Their expression levels were determined by the relative fluorescence units (RFU) of RFP fused with the enzymes. Significance levels of *t*-test: *P < 0.05, **P < 0.01.

A 7-DHC-producing strain SyBE_Sc0125XJ06, which was constructed in our previous study (7-DHC titer of 187.7 mg/L), was chosen as the starting strain for pathway compartmentation. In this strain, MVA pathway genes were all overexpressed under GAL promoters (Westfall et al., 2012) to enhance precursor supply (Guo et al., 2018). GAL promoters were functioned as inducible promoters during cultivation (Westfall et al., 2012) to separate the 7-DHC production stage from the cell growth stage. In strain SyBE_Sc0125XJ06, the genes *ERG5/ERG6* were knocked out to block branch metabolic flow to ergosterol, while one copy of gallus DHCR24, which was controlled by GAL1 promoter, was integrated into the *GAL1,7,10* locus (Johnston, 1987; Peng et al., 2017). As mentioned above, we have already known all the subcellular locations of the whole post-squalene proteins in *S. cerevisiae*, but not for DHCR24. DHCR24 is the only heterologous enzyme catalyzing one of the rate-limiting steps in the 7-DHC synthetic pathway. However, there is little research on gallus DHCR24 so far. According to Wu et al. (2004), research on human DHCR24, endogenous DHCR24, was located on the ER under basal conditions in human cells. The N-terminus of the human DHCR24 is identified as a potential transmembrane domain. This part of the sequences is predicted to partially embed in the membrane as a stem or “peduncle” rather than to traverse the membrane bilayer, which firmly anchors the protein to the membrane (Lu et al., 2012; Zerenturk et al., 2013). With a similar structure, when gallus DHCR24 (Caldwell et al., 2005) was introduced into *S. cerevisiae*, it would locate at a specific membrane structure with a high probability. However, yeasts have large and diverse membranes, making the issue on enzyme original location loss a possibility. Thus, we need to explore whether the relocating site of gallus DHCR24 in *S. cerevisiae* is still on the ER. Correspondingly, red fluorescent protein (RFP) was fused to the C-terminal of DHCR24 and coexpressed with green fluorescent protein (GFP)-linked Sec61, which was recognized as the specific marker for ER (Wilkinson et al., 1996; Kim et al., 2019). Confocal laser scanning microscopy demonstrated overlapped fluorescence signal patterns between DHCR24-RFP and Sec61-GFP (Figure 2A) proving that gallus DHCR24 is indeed located on the ER in yeast, which is consisted to DHCR24 in human cells.

As we illustrated above, the ERG protein distribution in yeast cells may cause an unbalanced metabolic flow. Since most of these proteins are mainly localized on the ER, we intended to express another copy on LDs to accelerate the delivering of intermediate metabolites. As reported, the protein oleosin from *Zea mays* (van Rooijen and Moloney, 1995; Mullner et al., 2004; Lin et al., 2017) and the N-terminal of a putative methyltransferase AAM-B (Zehmer et al., 2008) are commonly used peptides to direct the desired enzymes to yeast LDs. These two LD-targeting sequences are usually attached to the N-terminal of the target enzymes. We chose the yeast native enzymes ERG2/ERG3 and foreign enzyme DHCR24 to verify the function of these peptides especially on proteins with original transmembrane regions responding to ER location. As demonstrated in Figure 2B, oleosin was fused to ERG3, while AAM-B tag was fused to ERG2 and DHCR24, respectively. In order to verify whether these oleosin or AAM-B tag linked proteins were expressed in LDs, RFP was fused to their C-terminals. In the meanwhile, ERG7, which is the unique sterol synthesis enzyme that only localized on LDs, was selected as the LD-specific marker and expressed along with GFP fused to its C-terminal. Both the RFP and GFP fused proteins were co-expressed in the same cell. As a result, all the images of the red fluorescence signals were mainly coincident with the corresponding green ones (Figure 2B), suggesting that oleosin and AAM-B tags could target the ER proteins onto yeast LDs.

To test if relocation of enzymes could affect the protein expression levels, all the relocated enzymes and the original ER-located enzymes were fused with RFP, and their expression levels were determined by measuring the relative fluorescence units (RFU) of these enzymes. As shown in Figure 2C, fusing with the LD-relocating sequences did not improve protein expression of these relocated proteins, indicating that further increasing 7-DHC production by relocating post-squalene proteins to LDs were likely caused by other reasons.

### Compartmentalized Reconstitution of Downstream Post-squalene Pathway After Zymosterol

**FIGURE 3:**
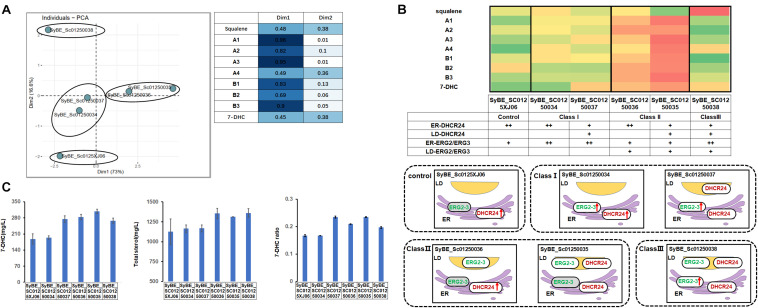
Compartmentalized reconstitution of downstream post-squalene enzymes. **(A)** Principal component analysis (PCA) of sterol intermediates of the strains under compartmentalized reconstitution of downstream ERG enzymes (after B1). Different classes of strains are circled by ellipses. The table shows the composition of the two principal components in the PCA experiment (Dim1 and Dim2). **(B)** The post-squalene pathway metabolic flux heat map. The accumulation levels of post-squalene intermediates were normalized according to Y = (X – μ)/σ, where Y represents the normalized value, X represents the original value, μ represents the mean value, and σ represents the standard deviation. The larger Y is presented by a color that is close to red, while the smaller one is close to green. The locations of engineered ERG proteins in the constructed strains are presented below the heat map. Backgrounds of the enzymes without modification are yellow. The up arrows represent protein overexpression. **(C)** 7-DHC productions, total sterols, and 7-DHC ratios of the constructed stains. All data were from at least triplicate experiments. *T*-test was conducted between the corresponding strain and the control strain SyBE_Sc0125XJ06. Significance levels of *t*-test: *P < 0.05, **P < 0.01.

Post-squalene pathway synthesizes subsequent sterols of squalene. Our former experiments showed that the main sterol intermediates accumulated in the post-squalene pathway are always distributed downstream of B1 (Figure 1B). These accumulated compounds also include B2 and lathosterol (B3). Sterols in the upstream pathway before B1 had little accumulation except squalene (Figure 1B). Therefore, the whole post-squalene pathway was divided into two parts: the pathway before B1 and the pathway after B1, and the pathway downstream of B1 (including B1) was optimized first. All the enzymes from this part were divided into two modules: ERG2/3 and DHCR24. Those modules were overexpressed either on ER or in LDs. To be specific, the control strain SyBE_0125XJ06 has one copy of the original ERG2/3 and two copies of DHCR24s expressed on ER. As shown in Figure 1D, we overexpressed ERG2/ERG3 by supplementing another ER- or LD-targeted copy in strain SyBE_0125XJ06, producing strain SyBE_01250034 or SyBE_01250036. Then one copy of the ER-targeted DHCR24 in strain SyBE_01250034 or SyBE_01250036 was replaced by the LD-targeted DHCR24, generating strain SyBE_01250037 or SyBE_01250035. One copy of ER-targeted ERG2/3 was further introduced into strain SyBE_Sc01250035, obtaining strain SyBE_Sc01250038. The production of 7-DHC and the accumulation of other metabolic intermediates from the post-squalene pathway in all these ERG2/3- or DHCR24-relocalized strains were evaluated by GC/MS after a 100-h shake-flask fermentation. In order to better explore the impact of the modification of strains on the 7-DHC metabolic pathways, we conducted the principal component analysis (PCA) on the data presented in Supplementary Table 1. Through PCA experiments, we calculated out two principal components, Dim1 and Dim2. Lanosterol (A1), 24,25-dihydrolanosterol (A2), 14-demethyllanosterol (A3), B1, B2, and B3 contributed mainly to Dim1, while squalene, 7-DHC, and 4,4-dimethyl-5alpha-cholesta-8-en-3beta-ol (A4) contributed both to Dim1 and Dim2 (Figure 3A). According to the PCA results (Figure 3A), all the tested strains were divided into three categories, i.e., Class I for SyBE_Sc01250034 and SyBE_Sc01250037, Class II for SyBE_Sc01250036 and SyBE_Sc01250035, and Class III for SyBE_Sc01250038. The differences among the strains belonging to these three classes depended on the location of ERG2/3, which might be the main cause of the changes in the metabolic flow of the three classes of strains, suggesting that the location of ERG2/3 was the key point in the modification of downstream proteins for improving 7-DHC production.

To be specific, in Class I, the strains have overexpressed ERG2/3, which were only located in the ER (Figure 3B). In the strains of this class, metabolites upstream of B1 were increased compared with the control strain in general, while the intermediate B2 was significantly reduced (Figure 3B). The decrease in B2 might be due to the enhanced catalytic efficiency of ER-located ERG2, and we also speculated that the increased accumulation of B3 might be brought by insufficient catalytic ability of ERG3. Compared with the control strain SyBE_Sc0125XJ06, the amounts of total sterols had no significant change, while only strain SyBE_Sc01250037, which harbors one copy of the LD-expressed DHCR24, achieved significant improvement on both 7-DHC production (by 46.1% to 274.2 mg/L) as well as 7-DHC ratio (increased by 40.7%) (Figure 3C). The increase in 7-DHC output came from the promoted conversion of sterol intermediates by targeting DHCR24 into LDs. That function of LD-targeted DHCR24 has also been proved by strains and SyBE_Sc01250035 from Class II.

In Class II the strains harbor another copy of LD-expressed ERG2/3 (Figure 3B). Most metabolites in the post-squalene pathway had been enhanced except squalene in strain SyBE_Sc01250035 (Figure 3B). The 7-DHC production of these two strains were both increased by 51.0% (to 283.4 mg/L) and 64.2% (to 308.2 mg/L), respectively (Figure 3C). In the meanwhile, their 7-DHC ratios were improved by 25.1 and 40.7%, respectively (Figure 3C). These data revealed that targeting ERG2/3 into LDs would not only improve the whole metabolic flux of the post-squalene pathway but also would be a benefit for the transformation of sterol intermediates into 7-DHC. The latter effect could be further enhanced by LD-targeted DHCR24.

Though 7-DHC production of strain SyBE_Sc01250035 increased most in the first part of our study, the intermediates like B2 and B3 also accumulated on a large scale. Thus, we introduced ER-located ERG2 and ERG3 to reduce B2 and B3. Thus, in Class III, the strain has overexpressed ER-located ERG2/3 as well as one copy of LD-targeted ERG2/3 (Figure 3B). Fermentation results showed that B2 and B3 of strain SyBE_Sc01250038 decreased sharply compared with strain SyBE_Sc01250035 as were our expectations. Among those post-squalene metabolites, only squalene and A4 were significantly accumulated and higher than those in the control strain. The accumulation of squalene was raised out of our expectation (Figure 3B), which was detrimental to 7-DHC production. Compared with the control strain, even though the 7-DHC ratio as well as the 7-DHC production were enhanced by 18.0%, and 42.7% (to 267.8 mg/L), respectively, the 7-DHC output was lower than strain SyBE_Sc01250035 without another copy of the ER-targeted ERG2/3 (Figure 3C). In addition, the Class III strain also took no advantage over strain SyBE_Sc01250035 on both total sterol accumulation and 7-DHC ratio, suggesting that further overexpressing ER-targeted ERG2/3 brought no benefit to 7-DHC synthesis. Therefore, strain SyBE_Sc01250035 was selected as the target for the next round of optimization.

### Compartmentalized Reconstitution of Upstream Post-squalene Pathway Before Zymosterol

A similar strategy was conducted on the pathway upstream of B1. ER-located post-squalene enzymes upstream of B1 were divided into two parts according to their catalytic order in the pathway, i.e., module ERG1/11/24 and module ERG25/26/27. Those modules were overexpressed either in the ER or in LDs (Figure 1D). To be specific, introducing one copy of ER- or LD-targeted ERG25/26/27 into strain SyBE_Sc01250035 generated strains SyBE01250039 and SyBE01250040, respectively, while introducing one copy of ER- or LD-targeted ERG1/11/24 into strain SyBE_Sc01250035 produced strains SyBE01250041 and SyBE01250042, respectively. Then a supplement of another copy of LD-expressed ERG25/26/27 into strains SyBE01250041 and SyBE01250042 obtained strains SyBE01250043 and SyBE01250044, respectively. These constructed strains were also cultivated for 100 h to measure their products by GC/MS.

**FIGURE 4:**
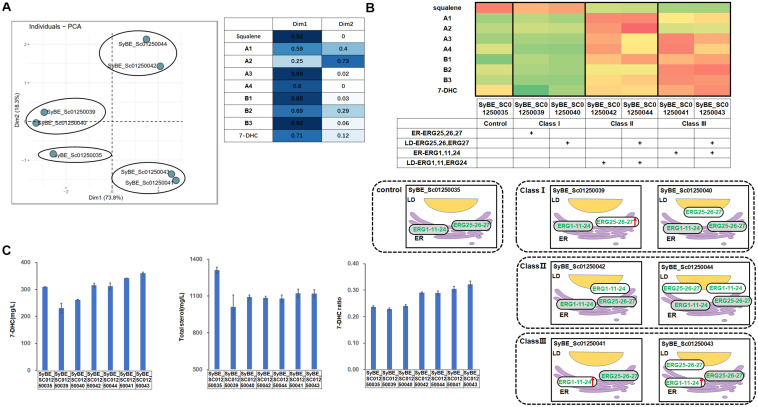
Compartmentalized reconstitution of upstream post-squalene enzymes. **(A)** PCA analysis of sterol intermediates of the strains under compartmentalized reconstitution of upstream ERG enzymes (before B1). Different classes of strains are circled by ellipses. The table shows the composition of the two principal components in the PCA experiment (Dim1 and Dim2). **(B)** The post-squalene pathway metabolic flux heat map. The accumulation levels of post-squalene intermediates were normalized according to Y = (X – μ)/σ, where Y represents the normalized value, X represents the original value, μ represents the mean value, and σ represents the standard deviation. The larger Y is presented by the color which is close to red, while the smaller one is close to green. The locations of engineered ERG proteins in the constructed strains are presented below the heat map. Backgrounds of the enzymes without modification are yellow. The up arrows represent protein overexpression. **(C)** 7-DHC productions, total sterols, and 7-DHC ratios of the constructed strains. All data were from at least triplicate experiments. *T*-test was conducted between the corresponding strain and the control strain SyBE_Sc0125XJ06. Significance levels of *t*-test: *P < 0.05, **P < 0.01, ***P < 0.001.

PCA experiments was also conducted on the data presented in Supplementary Table 2. Through PCA experiments, we calculated out two principal components, Dim1 and Dim2. Except A1, A2, and B2 contributing both to Dim1 and Dim2, other sterols like squalene, B3, A3, A4, etc., contributed most to Dim1. According to the results of PCA experiments (Figure 4A), all the tested strains were divided into three categories, i.e., Class I for SyBE_Sc01250039 and SyBE_Sc01250040, Class III for SyBE_Sc01250042, and SyBE_Sc01250044, as well as Class II for SyBE_Sc01250041 and SyBE_Sc01250043. The differences among the strains belonging to these three classes depended on whether ERG1/11/24 was overexpressed and where it was overexpressed, suggesting that the expression level as well as the location of ERG1/11/24 were the key points in the modification of upstream proteins for improving 7-DHC production.

In Class I, module ERG25/26/27 was overexpressed. As a result, no matter where ERG25/26/27 was located, there was still a dramatic accumulation of squalene (Figure 4B). Only A4 was sharply decreased (Figure 4B), due to overexpression of ERG25/26/27. Compared with the control strain SyBE_Sc01250035, the total sterol accumulation of SyBE_Sc01250040 as well as 7-DHC productions of strains SyBE_Sc01250039 and SyBE_Sc01250040 were both decreased, along with no improvement on the 7-DHC ratio.

In Class II, one copy of the LD-targeted ERG1/11/24 was introduced into the control strain SyBE_Sc01250035. Compared with the control strain, the accumulation of squalene declined dramatically by 78.9 and 81.1%, respectively, for strains SyBE_Sc01250042 and SyBE_Sc01250044. This part of squalene was mainly transformed into sterols upstream of B1 (Figure 4B). With unchanged 7-DHC productions and decreased total sterol amounts, 7-DHC ratios were improved by 22.9%.

Different from Class II, in Class III, one copy of ER-expressed ERG1/11/24 was introduced into the control strain SyBE_Sc01250035. Compared with the Class II strains, the accumulation of squalene in Class III strains was further reduced along with decreased amounts of upstream sterols A1 and A2 as well as increased production of downstream sterols B2, B3, and 7-DHC (Figure 4B), suggesting that ER-expressed ERG1/11/24, but not LD-targeted ERG1/11/24, could pull the metabolic flow from upstream squalene to the downstream sterols. Since either ER-expressed or LD-targeted ERG1/11/24 could reduce squalene accumulation, we speculated that ERG1 might function well on both organelles, but ERG11/24 might be more suitable in the ER than in LDs. As a result, even though the amounts of total sterols were also lower than that of the control strain, 7-DHC production of strains SyBE_Sc01250041 and SyBE_Sc01250043 increased by 10.5% (to 342.2 mg/L) and 16.4% (to 360.6 mg/L), respectively (Figure 4C), along with enhanced 7-DHC ratios. The improved 7-DHC titer and ratio of strain SyBE_Sc01250043 than SyBE_Sc01250041 was due to another copy of LD-expressed ERG25/26/27. Although this modification brought no positive effect in Class I strains, it reduced the accumulation of the substrates A3 and A4, and increased the production of B1 in both Class II and Class III strains (Figure 4B). It was deduced that overexpressing ERG25/26/27 in LDs converted more their precursors into downstream intermediates, which could help increase 7-DHC production in some strains like SyBE_Sc01250043. Strain SyBE_Sc01250043 achieved the highest 7-DHC production at shake-flask level as was known.

### Further Reducing the Metabolites of the Strain Modified by Compartmentalized Reconstitution

**FIGURE 5:**
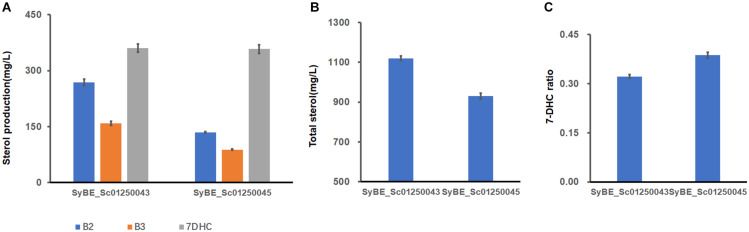
7-DHC production and metabolic flux changes caused by adding ER-targeted ERG2 and ERG3 to strain SyBE_Sc01250043. **(A)** B2, B3, and 7-DHC amounts change between SyBE_Sc01250043 and SyBE_Sc01250045. **(B)** Total sterols of the two strains. **(C)** 7-DHC ratios of the two strains. **(D)** The modified post-squalene pathway gene transcriptional levels in strain SyBE_Sc01250045 compared with the control strain SyBE_Sc0125XJ06. All data were from at least triplicate experiments. *T*-test was conducted between the corresponding strain and the control strain SyBE_Sc0125XJ06. Significance levels of *t*-test: *P < 0.05, **P < 0.01, ***P < 0.001.

According to our conclusions from a former part of the study, ER-located ERG2/3 could lower some sterols downstream of B1. In order to further reduce the accumulation of sterol metabolites, one copy of ER-expressed ERG2/3 was introduced into strain SyBE_Sc01250043, generating SyBE_Sc01250045. In strain SyBE_Sc01250045, 7-DHC had no significant change to strain SyBE_Sc01250043 (Figure 5A). Amounts of B2 and B3, the direct precursor of ERG2 and ERG3, both decreased dramatically (Figure 5A), and other sterols’ amounts had little change with strain SyBE_Sc01250043 (Supplementary Table 2). Therefore, the total sterol amounts of strain SyBE_Sc01250045 decreased (Figure 5B), and 7-DHC ratio increased, compared with those of strain SyBE_Sc01250043 (Figure 5C). The growth curves of all strains in this study were identical (Supplementary Figure 1). Also, the real-time quantitative PCR (QPCR) test results showed that transcription levels of most of the modified genes in strain SyBE_Sc01250045 (except *ERG1*, *ERG25*, and *DHCR24*) were increased compared with SyBE_Sc0125XJ06 (Figure 5D), which is one of the reasons for enhanced 7-DHC production. We speculated that the increased transcription levels of these genes may be due to the raised copy number of these genes. Besides, another reason for raised transcription levels may be enzymes relocated to the LDs. To be noticed, in the former study, when SyBE_Sc01250035 was introduced the same module (ER-located ERG2/3), the overall metabolic flux reduced, while only squalene increased in strain SyBE_Sc01250038. The reason why introducing the same ER-expressed ERG2/3 in different strains had a so different impact on metabolic flux still needs to be explained. In the meanwhile, since we presumed that ER-located ERG3 might have insufficient catalytic ability due to the increased accumulation of B3 in strains SyBE_Sc01250034 and SyBE_Sc01250037, other heterologous ERG3s should be screened and introduced into the 7-DHC-producing strain. In addition, a more delicate compartmentation test on those key modules like ERG2/3 and ERG1/11/24 should be also conducted by targeting a single ERG protein to one organelle at one time. Besides, we can further rearrange the LD-located enzymes like ERG7 to ER in the strains constructed in this study and investigate their effects on the accumulation of 7-DHC and other sterol intermediates.

## Conclusion

In this study, subcellular locations of post-squalene pathway enzymes were rearranged in order to improve the unbalanced metabolic flow and low product yield caused by the dual organelle locations. All the ER-located enzymes of the post-squalene pathway were grouped into four modules: ERG1/11/24, ERG25/26/27, ERG2/3, and DHCR24. These modules were overexpressed on ER or on LDs. We subsequently rearranged the locations of enzymes downstream and upstream of B1. We conducted PCA experiments to classify the strains and analyzed the metabolic flux of strains in each class. We also obtain some strains in which 7-DHC production and 7-DHC ratio both dramatically increased through rearranging the post-squalene pathway enzymes. Eventually, targeting modules ERG25/26/27 and ERG2/3 into LDs along with supplementing another copy of ER-located ERG1/11/24 promoted 7-DHC production from the initial 187.7–360.6 mg/L at shake-flask level in strain SyBE_SC01250043, while the squalene accumulation was dramatically reduced by 95.5% to 32.4 mg/L. Although adding another copy of ER-located ERG2/3 into strain SyBE_SC01250043 did not improve the 7-DHC titer, the 7-DHC ratio further increased to 39% of the total sterols, which is 2.32-fold of the initial ratio. This study achieves the highest shake-flask level titer of 7-DHC as was known, which demonstrated that compartmentalized reconstitution of the post-squalene pathway was an efficient way to optimize metabolic flux in the 7-DHC pathway and promote 7-DHC production and ratio.

## Materials and Methods

### Strains, Media, and Cultivation

All *E. coil* and *S. cerevisiae* strains used in this work are summarized in Table 1. All modified yeast strains were derived from CEN.PK2-1D. *E. coli* DH5α was used for molecular cloning. *E. coli* was grown in Luria broth (LB) medium containing 50 μg ml^–1^ of ampicillin or 50 μg ml^–1^ of kanamycin for construction of plasmids and strains. Solid synthetic complete (SC) medium (0.67% yeast nitrogen base, amino acid supplements, and 2% glucose) lacking the appropriate nutrient component were used to select recombinational yeast strains. Shake-flask fermentation was performed in modified YPD medium [2% peptone, 1% yeast extract, 4% glucose, and 1% D-(+)-galactose] at 30°C.

**TABLE 1:** *Saccharomyces cerevisiae* strains used in this study.

Strain	Description	Source
CEN.PK2-1D	*MAT*α, *URA3-52*, *TRP1-289*, *LEU2-3*,*112*, *HIS3*Δ*1*, *MAL2-8C*, *SUC2*	EUROSCARF
SyBE_Sc0125 × 001	CEN.PK2-1D, *LEU2:BieR-ERG19-*P_GAL1,10_*-ERG8*, *ADE1:tHMG1-*P_GAL1,10_*-IDI1_ADE1*, *HIS3:HIS3-ERG12-* P_GAL1,10_*-ERG10*, *URA3:tHMG1-* P_GAL1,10_*-ERG13-URA3*, *TRP1:tHMG1-* P_GAL1,10_*-ERG20-TPR1*, *GAL1,7, 10:HphR △ERG5 GAL7,10,1:*P_GAL1_*-Gg_DHCR24-*T_CYC1_	Previous study
SyBE_Sc0125XJ06	SyBE_Sc0125 × 001, *△ERG6:*P_GAL1_*-DHCR24-*T_CYC1_- *LEU2-*	Previous study
SyBE_Sc01250034	SyBE_Sc0125XJ06, *HO:*URA3-P_GAL1_-ERG3-T_FBA__1t_-P_GAL7_-ERG2-T_PGK__1t_	This study
SyBE_Sc01250035	SyBE_Sc0125 × 001, *△ERG6:*P_GAL1_*-AAMB-DHCR24-*T_CYC1_- *LEU2 HO:*URA3-P_GAL1_-Oleosin-ERG3-T_FBA__1t_-P_GAL7_-AAMB-ERG2-T_PGK__1t_	This study
SyBE_Sc01250036	SyBE_Sc0125XJ06, *HO:*URA3-P_GAL1_-Oleosin-ERG3-T_FBA__1t_-P_GAL7_-AAMB-ERG2-T_PGK__1t_	This study
SyBE_Sc01250037	SyBE_Sc0125 × 001, *△ERG6:*P_GAL1_*-AAMB-DHCR24-*T_CYC1_- *LEU2 HO:*URA3-P_GAL1_-ERG3-T_FBA__1t_-P_GAL7_-ERG2-T_PGK__1t_	This study
SyBE_Sc01250038	SyBE_Sc01250035, *Delta15:* P_GAL1_-ERG3-T_FBA__1t_-P_GAL7_-ERG2-T_PGK__1t_	This study
SyBE_Sc01250039	SyBE_Sc01250035, *Delta22:* P_GAL1_-ERG25-GGGGS-ERG26-T_FBA__1t_-P_GAL7_-ERG27-T_PGK__1t_	This study
SyBE_Sc01250040	SyBE_Sc01250035, *Delta22:*P_GAL1_-ERG25-Oleosin-ERG26-T_FBA__1t_-P_GAL7_-AAMB-ERG27-T_PGK__1t_	This study
SyBE_Sc01250041	SyBE_Sc01250035, *Delta22:*P_GAL1_-ERG1-GGGGS-ERG11-T_FBA__1t_-P_GAL7_- ERG24-T_PGK__1t_	This study
SyBE_Sc01250042	SyBE_Sc01250035, *Delta22:*P_GAL1_-ERG1-Oleosin-ERG11-T_FBA__1t_-P_GAL7_- AAMB-ERG24-T_PGK__1t_	This study
SyBE_Sc01250043	SyBE_Sc01250040, *Delta15:*P_GAL1_-ERG1-GGGGS-ERG11-T_FBA__1t_-P_GAL7_- ERG24-T_PGK__1t_	This study
SyBE_Sc01250044	SyBE_Sc01250040, *Delta15:*P_GAL1_-ERG1-Oleosin-ERG11-T_FBA__1t_-P_GAL7_- AAMB-ERG24-T_PGK__1t_	This study
SyBE_Sc01250045	SyBE_Sc01250043, *Tau3:*KANMX-P_GAL1_-ERG3-T_FBA__1t_-P_GAL7_-ERG2-T_PGK__1t_	This study
SyBE_Sc0125P001	CEN.PK2-1D, PRS425K- P_GAL1_*-Gg_DHCR24-RFP-*T_FBA1_, PRS416- P_*GAL*__1_*SEC61-GFP-*T_FBA1_	This study
SyBE_Sc0125P002	CEN.PK2-1D, PRS425K- P_GAL1_*-AAMB-DHCR24-RFP-*T_FBA1_, PRS416- P_*GAL*__1_*ERG7-GFP-*T_FBA1_	This study
SyBE_Sc0125P003	CEN.PK2-1D, PRS425K- P_GAL1_*-AAMB-ERG2-RFP-*T_FBA1_, PRS416- P_*GAL*__1_*ERG7-GFP-*T_FBA1_	This study
SyBE_Sc0125P004	CEN.PK2-1D, PRS425K- P_GAL1_*-Oleosin-ERG3-RFP-*T_FBA1_, PRS416- P_*GAL*__1_*ERG7-GFP-*T_FBA1_	This study

For shake-flask fermentation, constructed strains were cultured and purified on an SC plate. A single colony was cultivated in 5 ml of YPD medium overnight at 30°C, and the culture was transferred into 40 ml of modified YPD when cells entered the mid-exponential phase. The initial glucose concentration in YPD medium was 40 g/L and OD_600_ was 0.2. D-galactose (10 g/L) was added into the media at the beginning of fermentation for inducing GAL promoters of recombinational strains. Shake-flask fermentation lasted for 100 h.

### Construction of Plasmids and Strains

For constructing ERG2, ERG3, and DHCR24 relocalizing strains, *ERG2* and *ERG3* were fused with LD-targeting sequence AAM-B and oleosin to their 3’-terminuses individually by OE-PCR (Supplementary Figure 2). Modified genes *AAM-B-ERG2* and *oleosin-ERG3* were cloned into expression cassettes containing *GAL7* or *GAL1* promoter, which were built in our previous study. The original genes *ERG2* and *ERG3* were also cloned into the corresponding expression cassettes for control. Expression cassettes containing the desired genes “*TDH2t-GAL1p-gene1-FBA1t*” and “*FBA1t-GAL7p-gene2-PGK1t*” were then PCR amplified with 20-bp overlap to plasmid PRS425K and flanking specific restriction enzyme sites at one terminus, and the two expression cassettes also had a 20-bp overlap at their adjacent ends. The two resulting amplicons were assembled into linearized vector PRS425K with SE Seamless Cloning and Assembly Kit (Beijing Zoman Biotechnology). The recombinant modules containing two gene expression cassettes were released from plasmids by corresponding restriction enzymes cutting at the 5′ and 3′ terminus of the whole modules as genomic integration fragment (no. 1). Genomic sequences covering around 700 bp upstream of the desired integration site, selection marker, and terminator TDH2t (no selection marker if the integration was in the CRISPR method, when *ERG2* and *ERG3* were integrated in the *YPRC*Δ*15* locus) were amplified from *S. cerevisiae* genome and joined by overlap PCR. The resulting amplicon was ligated into a PEAZY-blunt vector, and the recombinant plasmids were digested by restriction enzymes to yield the left arm fragment of the homologous recombination (no. 2). The right arm fragment (no. 3) was obtained in the same way as terminator PGK1t and 700-bp downstream of the desired integration locus. The three fragments above were transformed using the LiAc/SS carrier DNA/PEG method. For integration of *ERG2/ERG3* in *YPRC*Δ*15* locus, the CRISPR method was used. Two complementary primers from the target sequence *YPRC*Δ*15* were annealed and ligated into CRISPR plasmid (carrying *cas9* and incomplete gRNA), generating the complete guide RNA. The resulting CRISPR plasmid was transformed with the three recombination fragments above. The DHCR24 expression module and the left/right arm fragments of homologous recombination were obtained from our previous study and integrated into genome *ERG6* locus in a similar way. For constructing ERG1, ERG11, ERG24, ERG25, ERG26, and ERG27 overexpressing or relocalizing strains, the similar molecular manipulation strategy was applied except two differences: (1) According to Lin et al. (2017), oleosin sequence could relocalize two proteins into the LDs simultaneously when proteins fused to its 5′-and 3′-terminus. *ERG1*, oleosin, and *ERG11* were amplified and combined by OE-PCR (Supplementary Figure 1), the resulting amplicons were recombined into the *GAL1p* expression cassette. In order to be consistent with the relocalization method, ERG1 and ERG11 used DNA linker GGGGS to became a fused protein for their overexpression experiment. ERG25 and ERG26 overexpressing or relocalizing strains were also constructed in the same way. (2) The right arm fragment of the homologous recombination for *ERG* genes integrating upstream of B1 was different. Expression cassette “*FBA1t-GAL7p-gene-PGK1t*” and about 700 bp of overlap of downstream, the desired genomic integration site was joined by OE-PCR as the right arm fragment to reduce the number of recombination fragments to three.

### Extraction and Analysis of Sterols

Sterol extraction was performed as previously described (Guo et al., 2018). Yeast cells were harvested by centrifugation at 12,000 rpm for 2 min after fermentation process and boiled in 3 N HCl for 5 min to break the cell wall. Cells were pelleted and washed by distilled water to remove the remaining HCl. After neutralizing by NaOH, saponification reaction of cells was carried out in 3 M NaOH-methanol solution at 60°C for 4 h. n-Hexane and silica sand were added for sterol extraction with vortex. The n-hexane phase was collected and dried by a centrifugal vacuum evaporator. N-methyl-N-(trimethylsilyl) trifluoroacetamide (MSTFA) was used to derivatize the obtained sterols (30°C for h) and samples were ready for GC-MS analysis.

Sterols were separated and analyzed by GCMS-QP2020 (SHIMADZU, Japan) using a DB-5 fused-silica capillary column (30 m × 0.25 mm i.d., film thickness 0.25 μm, J&W Scientific, CA). Mass spectra ranged at 50–800 m/z, and helium was used as the carrier gas. Operating conditions were inlet temperature 260°C, initial temperature 70°C for 2 min then ramp 30°C/min to 250°C then ramp 10°C/min to 280°C and held for 15 min. Finally, the temperature increased to 290°C at 5°C/min and was held for 5 min. Sterol standards (squalene, A1, A2, B3, B1, 7-DHC, etc.) were purchased from Sigma-Aldrich (United States).

### Principal Component Analysis

All visualizations of principal component analysis (Pearson, 2010) were performed in the R package FactoMineR (Lê et al., 2008).

### Protein Expression Level Analysis

Strains were cultured in SC-leu medium with 20 g/L of galactose for 60 h. A plate reader (SpectraMAX M2, Molecular Devices) used with a 587-nm excitation filter and a 610-nm emission filter was for measurement of the fluorescence of RFP.

### Genes Transcriptional Analysis

The real-time quantitative PCR (QPCR) process was similar to our published article (Guo et al., 2018). Strains were cultured in a shake flask for 30 h (ethanol consumption phase) and then harvested. Total RNA extraction, reverse transcription, and QPCR were carried out by Apexbio Inc. (China). The gene *ALG9* was used for normalization.

## Data Availability Statement

The original contributions presented in the study are included in the article/Supplementary Material, further inquiries can be directed to the corresponding author/s.

## Author Contributions

X-JG and YW conceived the study as well as participated in the strain construction and carried out the molecular genetic studies. X-JG and W-HX conducted the fermentation. M-DY, G-RZ, and Y-JY participated in the design and coordination of the study as well as helped draft the manuscript. YW supervised the whole research and revised the manuscript. All authors read and approved the final manuscript.

## Conflict of Interest

The authors declare that the research was conducted in the absence of any commercial or financial relationships that could be construed as a potential conflict of interest.
