# Supplementary material for: Compartmentalized Reconstitution of Post-squalene Pathway for 7-Dehydrocholesterol Overproduction in Saccharomyces cerevisiae
Source: Front Microbiol. 2021 May 21;12:663973. doi: 10.3389/fmicb.2021.663973 (PMC8175624; doi:10.3389/fmicb.2021.663973)
Supplement: Supplementary file 1 [file Data_Sheet_1.docx]

## Compartmentalized reconstitution of post-squalene pathway for 7-dehydrocholesterol overproduction in *Saccharomyces cerevisiae*

**Xiao-jing Guo^1, 2^, Ming-Dong Yao^1, 2^, Wen-Hai Xiao^1, 2^, Ying Wang^1, 2^*, Guang-Rong Zhao^1,2^, Ying-Jin Yuan^1, 2^**

^1^ Frontier Science Center for Synthetic Biology and Key Laboratory of Systems Bioengineering (Ministry of Education), School of Chemical Engineering and Technology, Tianjin University, Tianjin 300072, China

^2^ Collaborative Innovation Center of Chemical Science and Engineering (Tianjin), Tianjin University, Tianjin 300072, China

***Correspondence:**
Ying Wang
Email: [ying.wang@tju.edu.cn](mailto:ying.wang@tju.edu.cn)

**
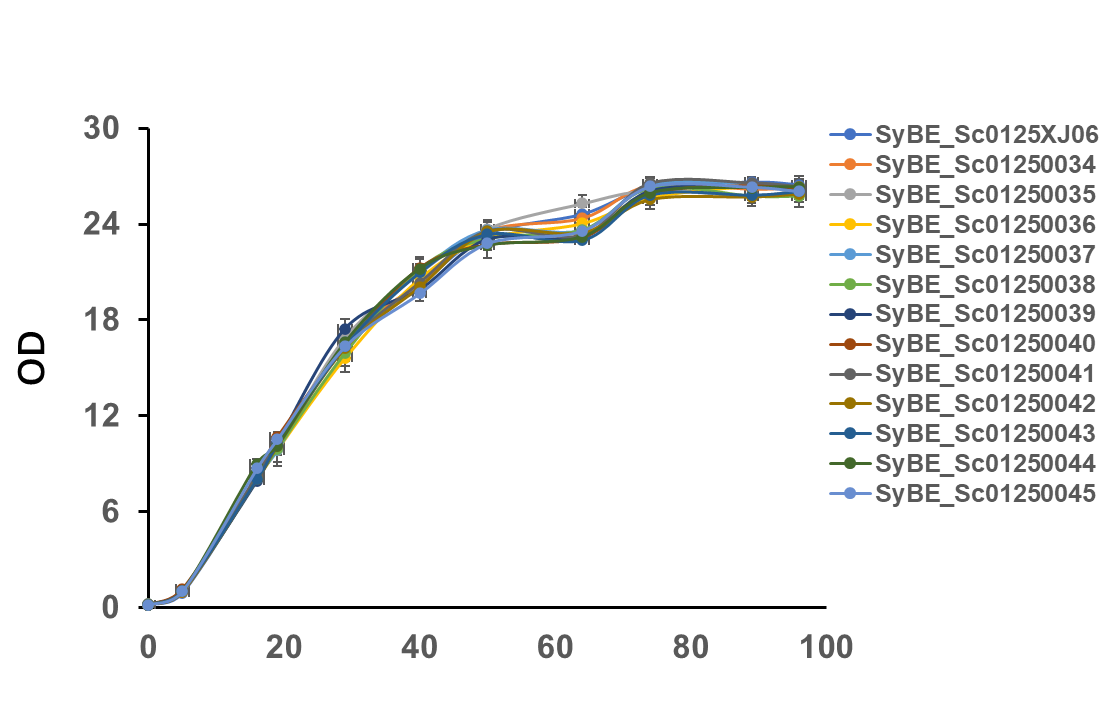
**

**Supplementary Figure 1.** The growth curves of each strain in this study.


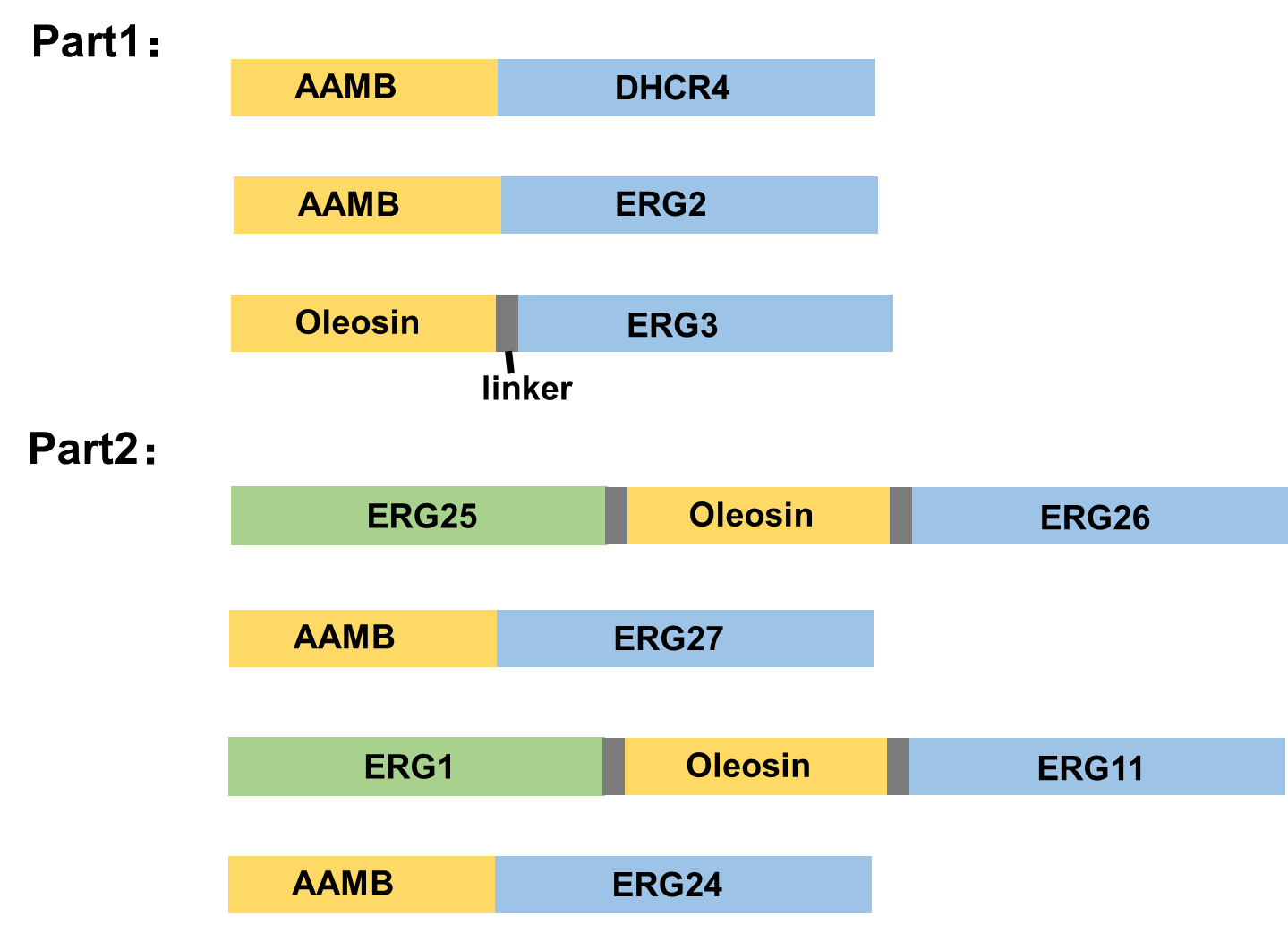


**Supplementary Figure 2.** Schematic representation of construction method for compartmentalized reconstitution of pathway downstream zymosterol (B1) (Part1) and upstream zymosterol (B1) (Part2).

**Supplementary Table S1.** **Accumulation of** **sterol intermediates of** **strains under compartmentalized reconstitution of ERG-enzymes downstream zymosterol (B1).**

Unit: mg/L

|  | SyBE_Sc0125XJ06 | SyBE_Sc01250034 | SyBE_Sc01250037 | SyBE_Sc01250036 | SyBE_Sc01250035 | lSyBE_Sc01250038 |
| --- | --- | --- | --- | --- | --- | --- |
| squalene | 718.89±80.71 | 764.84±12.83 | 700.98±21.9 | 750.27±30.3 | 617.49±22.25 | 945.88±20.79 |
| Lanosterol (A1) | 2.64±0.06 | 4.19±0.01 | 4.15±1.36 | 5.36±0.67 | 6.67±0.34 | 2.97±0.55 |
| 24,25-Dihydrolanosterol (A2) | 30.26±1.66 | 32.77±0.5 | 29.99±2.82 | 41.77±3.27 | 42.34±0.75 | 20.92±1.6 |
| 14-Demethyllanosterol (A3) | 0.86±0.23 | 1.2±0.29 | 1.65±0.32 | 1.86±0.72 | 2.66±0.58 | 0.89±0.05 |
| 4,4-Dimethyl-5alpha-cholesta-8-en-3beta-ol (A4) | 10.39±0.98 | 20.51±0.5 | 16.85±1.03 | 20.51±0.88 | 29.21±0.21 | 21.71±1.06 |
| Zymosterol (B1) | 52.05±1.96 | 63.25±9.62 | 59.16±4.52 | 71.89±0.67 | 92.53±3.22 | 23.33±6.47 |
| 5alpha-Cholest-8-en-3beta-ol (B2) | 83.31±11.01 | 39.3±2.47 | 36.8±0.73 | 107.31±6.04 | 128.05±5.85 | 31.56±1.42 |
| Lathosterol (B3) | 38.37±5.66 | 44.74±15.82 | 44.23±3.9 | 72.57±3.61 | 86.58±0.21 | 46.03±1.9 |
| 7-DHC | 187.72±22.42 | 194.21±7.39 | 274.16±14.42 | 283.4±12.25 | 308.2±9.69 | 267.79±11.29 |

**Supplementary Table S2. Accumulation of sterol intermediates of strains under compartmentalized reconstitution of ERG-enzymes upstream zymosterol (B1).**

Unit: mg/L

|  | SyBE_Sc01250035 | SyBE_Sc01250039 | SyBE_Sc01250040 | SyBE_Sc01250042 | SyBE_Sc01250044 | SyBE_Sc01250041 | SyBE_Sc01250043 | SyBE_Sc01250045 |
| --- | --- | --- | --- | --- | --- | --- | --- | --- |
| squalene | 620.21±13.1 | 438.9±57.04 | 509.59±12.21 | 131.09±13.73 | 117.29±2.54 | 38.2±5.45 | 32.42±14.42 | 38.76±4.89 |
| Lanosterol (A1) | 5.66±0.3 | 7.26±0.9 | 5.96±0.02 | 19.8±0.79 | 19.93±1.1 | 11.55±1.54 | 12.92±4.3 | 12.54±0.25 |
| 24,25-Dihydrolanosterol (A2) | 42.94±0.93 | 49.9±2.04 | 47.03±4.25 | 112.51±7.93 | 137±5.98 | 53.46±2.49 | 55.99±4.57 | 59.23±2.69 |
| 14-Demethyllanosterol (A3) | 2.38±0.06 | 2.75±0.11 | 2.44±0.41 | 17.78±3.26 | 10.04±1.35 | 22.26±2.09 | 17.35±2.79 | 15.85±0.25 |
| 4,4-Dimethyl-5alpha-cholesta-8-en-3beta-ol (A4) | 28.71±0.92 | 9.69±1.88 | 8.16±1.79 | 71.84±17.13 | 40.99±1.95 | 82.29±7.13 | 50.71±7.71 | 50.3±5.28 |
| Zymosterol (B1) | 90.14±4.26 | 106.19±21.46 | 100.29±10.23 | 148.77±24 | 163.31±9.01 | 153.01±11.06 | 162.75±14.74 | 171.91±19.44 |
| 5alpha-Cholest-8-en-3beta-ol (B2) | 126.65±6.32 | 95.52±8.82 | 88.83±0.19 | 147.62±5.51 | 148.22±2.96 | 255.08±28.63 | 268.36±27.1 | 134.45±1.77 |
| Lathosterol (B3) | 84.32±1.25 | 69.03±16.55 | 66.86±11.88 | 120.29±3.81 | 129.29±1.81 | 163.87±14.93 | 158.96±25.62 | 87.98±1.39 |
| 7-DHC | 309.69±0.32 | 231.37±16.73 | 261.87±1.81 | 315.8±6.67 | 312.34±11.75 | 342.2±0.41 | 360.61±3.98 | 358.2±10.77 |

**Supplementary Table S3.** Oligonucleotides used in this study.

| **Oligos** | **Sequence (5’- 3’)** |
| --- | --- |
| **For construction of module *gal1p-sec61-GGGGS-GFP-FBA1t*** | |
| gal1p_F | ATAAGCTTGATATCGAATTCACGGATTAGAAGCCGCCGAGC |
| gal1p_R | AGAACACGGTTGGAGGACATTTATAGTTTTTTCTCCTTGACGTTAAAG |
| sec61_F | TCAAGGAGAAAAAACTATAAATGTCCTCCAACCGTGTTC |
| Sec61_R | CCTTTAGATGAACCACCACCCATCAAATCAGAAAATCCTGG |
| GFP_F | CAGGATTTTCTGATTTGATGGGTGGTGGTTCATCTAAAGGTG |
| GFP_R | TCAATTAATTTGAATTAACTTTATTTGTACAATTCATCCATACCATG |
| FBA1t_F | TGGATGAATTGTACAAATAAAGTTAATTCAAATTAATTGATATAGTTTTTTAATG |
| FBA1t_R | CCGCTCTAGAACTAGTGGATCCAAAGATGAGCTAGGCTTTTGTAAAAATATC |
| **For construction of module *ERG7-GGGGS-GFP*** | |
| ERG7_F | TCAAGGAGAAAAAACTATAAATGACAGAATTTTATTCTGACACAATC |
| GFP_R | TCAATTAATTTGAATTAACTTTATTTGTACAATTCATCCATACCATG |
| **For construction of module *DHCR24-GGGGS-RFP*** | |
| DHCR24_F | AAAGGTCTCCAATGAGTGCCGTTTGGTCTTTAG |
| DHCR24_R | TTCACCTTTTGAAACAGAACCACCACCACCATGTCTAGCGGCTTTGCAAATC |
| RFP_F | AAAGCCGCTAGACATGGTGGTGGTGGTTCTGTTTCAAAAGGTGAAGAAGATAATATG |
| RFP_R | ACAGGTCTCGTTTATTTATATAATTCATCCATACCACC |
| **For construction of module AAMB-*DHCR24-GGGGS-RFP*** | |
| AAMB-DHCR24_F | AAAGGTCTCCAATGGAGTTGACAATTTTTATTTTAAG |
| AAMB-DHCR24_R | TTCACCTTTTGAAACAGAACCACCACCACCATGTCTAGCGGCTTTGCAAATC |
| RFP_F | AAAGCCGCTAGACATGGTGGTGGTGGTTCTGTTTCAAAAGGTGAAGAAGATAATATG |
| RFP_R | ACAGGTCTCGTTTATTTATATAATTCATCCATACCACC |
| **For construction of module *AAMB-ERG2-GGGGS-RFP*** | |
| AAMB28-ERG2_F | AAAGGTCTCCAATGGAGTTGACAATTTTTATTTTAAG |
| AAMB28-ERG2_R | GAAACAGAACCACCACCACCGAACTTTTTGTTTTGCAACAAG |
| RFP_F | AACTTGTTGCAAAACAAAAAGTTCGGTGGTGGTGGTTCTGTTTC |
| RFP_R | ACAGGTCTCGTTTATTTATATAATTCATCCATAC |
| **For construction of module *Oleosin-ERG3-GGGGS-RFP*** | |
| Oleosin_F | TCAAGGAGAAAAAACTATAAATGGCTGATAGAGATAGGTCTGG |
| Oleosin_R | GCGACTTCTAAGACCAAATCGCTAGAGGCTCTTCCACCGC |
| ERG3_F | GCGGTGGAAGAGCCTCTAGCGATTTGGTCTTAGAAGTCGCTG |
| ERG3_R | GAAACAGAACCACCACCACCGTTGTTCTTCTTGGTATTTGGG |
| RFP_F | CAAATACCAAGAAGAACAACGGTGGTGGTGGTTCTGTTTC |
| RFP_R | TATATCAATTAATTTGAATTAACTTTATTTATATAATTCATCCATACCACC |
| **Primers for *ERG2* amplification** | |
| ERG2_F | AAAGGTCTCCAATGAAGTTTTTCCCACTCCTTTTG |
| ERG2_R | ACAGGTCTCGTTTAGAACTTTTTGTTTTGCAACAAG |
| **primers for *ERG3* amplification** | |
| ERG3_F | TCAAGGAGAAAAAACTATAAATGGATTTGGTCTTAGAAGTCG |
| ERG3_R | TCAATTAATTTGAATTAACTTTAGTTGTTCTTCTTGGTATTTGG |
| **Primers for AAM-B-ERG2** | |
| AAMB_F | AAAGGTCTCCAATGGAGTTGACAATTTTTATTTTAAG |
| AAMB_R | AAAAGGAGTGGGAAAAACTTATATGGAAACCACTTCTTGCATATC |
| ERG2_F | GCAAGAAGTGGTTTCCATATAAGTTTTTCCCACTCCTTTTG |
| ERG2_R | ACAGGTCTCGTTTAGAACTTTTTGTTTTGCAACAAG |
| **Primers for AAM-B-ERG3** | |
| AAMB_F | AAACGTCTCCAATGGAGTTGACAATTTTTATTTTAAG |
| AAMB_R | GCGACTTCTAAGACCAAATCATATGGAAACCACTTCTTGCATATC |
| ERG3_F | GCAAGAAGTGGTTTCCATATGATTTGGTCTTAGAAGTCGCTG |
| ERG3_R | ACACGTCTCGTTTAGTTGTTCTTCTTGGTATTTGG |
| **Primers for *AAM-B-DHCR24*** | |
| AAMB_F | AAAGGTCTCCAATGGAGTTGACAATTTTTATTTTAAG |
| AAMB_R | CCTAAAGACCAAACGGCACTATATGGAAACCACTTCTTGCATATC |
| DHCR24_F | GCAAGAAGTGGTTTCCATATAGTGCCGTTTGGTCTTTAGG |
| DHCR24_R | ACAGGTCTCGTTTAATGTCTAGCGGCTTTGC |
| **For construction of module *TDH2t-GAL1p-ERG3(Olesin-ERG3)-FBA1t-ERG2(AAMB-ERG2)-PGK1t*** | |
| PRS-TDH2T_F | ATAAGCTTGATATCGAATTCAAAACTGCAGGCGGCCGCATTTAAC |
| FBA1T-ERG3_R | ATCAATTAATTTGAATTAACTTAGTTGTTCTTCTTGG |
| ERG3-FBA1T_F | GTTAATTCAAATTAATTGATATAGTTTTTTA |
| PRS-PGK1T_R | GCGGCCGCTCTAGAACTAGTCGCGGATCCGCGGCCGCAAC |
| **For construction of module HO_L-URA-TDH2t** | |
| HO_L-TDH2t_F | AAAAGCGGCCGCACGTGTGTGTCTCATGGAAATTG |
| HO_L-TDH2t-R | TGAATTGAATTGAAAAGCTTTTTAAAGTATAGATAGAATTGATTGCTG |
| URA_F | AATTCTATCTATACTTTAAAAAGCTTTTCAATTCAATTCATC |
| URA_R | AGTAACTTAAGGAGTTAAATCCCGGGTAATAACTGATATAATTAAATTG |
| TDH2t_F | TATATCAGTTATTACCCGGGATTTAACTCCTTAAGTTACTTTAATGATTTAG |
| TDH2t_R | CGCGGATCCGCGAAAAGCCAATTAGTGTG |
| **For construction of module PGK1t-HO_R** | |
| PGK1t _F | AAAACTGCAGGCGGCCGCATTGAATTGAATTGAAATCGATAG |
| PGK1t _R | TTTAAACTAATATACACATTAACGAACGCAGAATTTTCGAG |
| HO_R_F | TCGAAAATTCTGCGTTCGTTAATGTGTATATTAGTTTAAAAAGTTGTATG |
| HO_R_R | CGCGGATCCGCGGCCGCTCTAACATCACTATCTCTAACGCTG |
| **For construction of module delta15_L-TDH2t** | |
| delta15_L_F | AAAACTGCAGGCGGCCGCGCATACTCACTATCGTAAACTGTCC |
| delta15_L_R | AGTAACTTAAGGAGTTAAATATTTTATTACGTCATATATGAATACTAGTCAATAG |
| TDH2t_F | CATATATGACGTAATAAAATATTTAACTCCTTAAGTTACTTTAATGATTTAG |
| TDH2t_R | CGCGGATCCGCGGCCGCGCGAAAAGCCAATTAGTGTG |
| **For construction of module PGK1t-delta15_R** | |
| PGK1t_F | AAAACTGCAGGCGGCCGCATTGAATTGAATTGAAATCGATAG |
| PGK1t_R | CGTTATTTAACGAATTTATTAACGAACGCAGAATTTTCG |
| delta15_R_F | TCGAAAATTCTGCGTTCGTTAATAAATTCGTTAAATAACGGTGTG |
| delta15_R_R | CGCGGATCCGCGGCCGCCCTTTTTGCACAGTTAAACTACC |
| **Annealing primers for delta15 target sequence** | |
| YPRCdelta15-gRNA-F | GACTTTATATGTTTGGTTTCGATTGT |
| YPRCdelta15-gRNA-R | AAACACAATCGAAACCAAACATATAA |
| **For construction of module *ERG1-GGGGS-ERG11*** | |
| ERG1-GGGGS_F | TCAAGGAGAAAAAACTATAAATGTCTGCTGTTAACGTTGCAC |
| ERG1-GGGGS_R | GCAGAAGAACCACCACCACCACCAATCAACTCACCAAACAA |
| GGGGS-ERG11_F | TGTTTGGTGAGTTGATTGGTGGTGGTGGTGGTTCTTCTGCTACCAAGTCAATCGTTG |
| GGGGS-ERG11_R | TCAATTAATTTGAATTAACTTTAGATCTTTTGTTCTGGATTTCTC |
| **For construction of module *ERG1-Oleosin-ERG11*** | |
| ERG1_F | TCAAGGAGAAAAAACTATAAATGTCTGCTGTTAACGTTGCAC |
| ERG1_R | GCCATTGATCCTCCTCCTCCACCAATCAACTCACCAAACAAAAATG |
| OLE_F | TGTTTGGTGAGTTGATTGGTGGAGGAGGAGGATCAATGG |
| OLE_R | ACGATTGACTTGGTAGCAGAAGAACCACCACCACCGCTAG |
| ERG11_F | CTAGCGGTGGTGGTGGTTCTTCTGCTACCAAGTCAATCGTTG |
| ERG11_R | TCAATTAATTTGAATTAACTTTAGATCTTTTGTTCTGGATTTCTC |
| **Primers for *ERG24* amplification** | |
| ERG24_F | GTTGAATATTCCCTCAAAAAATGGTATCAGCTTTGAATCCC |
| ERG24_R | GATTTCAATTCAATTCAATTTTAATAAACATATGGAATGATCTTGTAAG |
| **Primers for AAM-B-ERG24** | |
| AAMB_F | GTTGAATATTCCCTCAAAAAATGGAGTTGACAATTTTTATTTTAAG |
| AAMB_R | CTGGGATTCAAAGCTGATACATATGGAAACCACTTCTTGCATATC |
| ERG24_F | GCAAGAAGTGGTTTCCATATGTATCAGCTTTGAATCCCAGAAC |
| ERG24_R | GATTTCAATTCAATTCAATTTTAATAAACATATGGAATGATCTTGTAAG |
| **For construction of module *FBA1t-GAL7p-ERG24(ERG27)-PGK1t-delta22_R* or *FBA1t-GAL7p-AAM-B-ERG24(27)-PGK1t-delta22_R*** | |
| FBA1t-GAL7p-ERG24(ERG27)-PGK1t_F | TTGATATCGAATTCCTGCAGGCGGCCGCGTTAATTCAAATTA |
| FBA1t-GAL7p-ERG24(ERG27)-PGK1t_R | AGAGTGAATAGTAATATTTTAACGAACGCAGAATTTTCGAG |
| delta22_R_F | TCGAAAATTCTGCGTTCGTTAAAATATTACTATTCACTCTGCTAAGATTATC |
| delta22_R_R | GCTCTAGAACTAGTGGATCCGCGGCCGCGGGCTTGTCGTGATAAACGATC |
| **For construction of module *delta22_L-TDH2t*** | |
| delta22_L_F | AAAACTGCAGGCGGCCGCCGTTGGAGGCAGAAACAATTTTG |
| delta22_L_R | AGTAACTTAAGGAGTTAAATTATAAGCAGTCTTGGGGAAGCAAC |
| TDH2t_F | CTTCCCCAAGACTGCTTATAATTTAACTCCTTAAGTTACTTTAATGATTTAG |
| TDH2t_R | CGCGGATCCGCGGCCGCGCGAAAAGCCAATTAGTGTGATAC |
| **For construction of module *ERG25-GGGGS-ERG26*** | |
| ERG25_F | TCAAGGAGAAAAAACTATAAATGTCTGCCGTTTTCAACAAC |
| ERG25_R | TTTGAAGAACCACCACCACCGTTAGTCTTCTTTTGAGCATTGTTTTC |
| ERG26_F | ATGCTCAAAAGAAGACTAACGGTGGTGGTGGTTCTTCAAAG |
| ERG26_R | TCAATTAATTTGAATTAACTTTACAAACCTTCGTCCATCC |
| **For construction of module *ERG25-Oleosin-ERG26*** | |
| ERG25_F | TCAAGGAGAAAAAACTATAAATGTCTGCCGTTTTCAACAAC |
| ERG25_R | GACCTATCTCTATCAGCCATTGATCCTCCTCCTCCGTTAGTCTTCTTTTGAGCATTGTTTTC |
| Oleo_F | CTAACGGAGGAGGAGGATCAATGGCTGATAGAGATAGGTCTGG |
| Oleo_R | AAAACTGAATCTATCTTTGAAGAACCACCACCACCGCTAGAGGCTCTTCCACCGC |
| ERG26_F | CTAGCGGTGGTGGTGGTTCTTCAAAGATAGATTCAGTTTTAATTATCG |
| ERG26_R | TCAATTAATTTGAATTAACTTTACAAACCTTCGTCCATCCAG |
| **Primers for *ERG27* amplification** | |
| ERG27_F | GTTGAATATTCCCTCAAAAAATGAACAGGAAAGTAGCTATCGTAAC |
| ERG27_R | GATTTCAATTCAATTCAATTTTAAATGGGGGTTCTAGTTTCAAC |
| **Primers for AAM-B-ERG27** | |
| AAMB_F | GTTGAATATTCCCTCAAAAAATGGAGTTGACAATTTTTATTTTAAG |
| AAMB_R | ACGATAGCTACTTTCCTGTTATATGGAAACCACTTCTTGCATATC |
| ERG27_F | GCAAGAAGTGGTTTCCATATAACAGGAAAGTAGCTATCGTAACG |
| ERG27_R | GATTTCAATTCAATTCAATTTTAAATGGGGGTTCTAGTTTCAAC |
| **Annealing primers for delta22 target sequence** | |
| delta22-gRNA_F | GACTTTGAACCTTTCCCCGAGTATGT |
| delta22-gRNA_R | AAACACATACTCGGGGAAAGGTTCAA |
| **For construction of module *FBA1t-GAL7p-ERG24(ERG27)-PGK1t-delta15_R* or *FBA1t-GAL7p-AAM-B-ERG24(27)-PGK1t-delta15_R*** | |
| FBA1t-GAL7p-ERG24(ERG27)-PGK1t_F | TTGATATCGAATTCCTGCAGGCGGCCGCGTTAATTCAAATTA |
| FBA1t-GAL7p-ERG24(ERG27)-PGK1t_R | CGTTATTTAACGAATTTATTAACGAACGCAGAATTTTCGAG |
| delta15_R_F | TCGAAAATTCTGCGTTCGTTAATAAATTCGTTAAATAACGGTGTG |
| delta15_R_R | GCTCTAGAACTAGTGGATCCGCGGCCGCCCTTTTTGCACAG |
| **For construction of module *Tau3_L-KANMX-TDH2t*** | |
| tau3_L_F | GCGGCCGCCGATAGCTGCGTTGTTGTTGAAG |
| tau3_L_R | ATTCTGGGCCTCCATGTCGCCAACAACCTCTTGCTATCAAAC |
| KanMX_F | TTGATAGCAAGAGGTTGTTGGCGACATGGAGGCCCAGAATACCC |
| KanMX_R | AGTAACTTAAGGAGTTAAATTTAGAAAAACTCATCGAGCATCAAATG |
| TDH2_F | TGCTCGATGAGTTTTTCTAAATTTAACTCCTTAAGTTACTTTAATGATTTAG |
| TDH2t_R | GCGGCCGCGCGAAAAGCCAATTAGTGTGATAC |
| **For construction of module *PGK1t- Tau3_R*** | |
| PGK1t_F | AAAACTGCAGGCGGCCGCATTGAATTGAATTGAAATCGATAG |
| PGK1t_R | TGGAGAGATATATTCTAAAAAACGAACGCAGAATTTTCGAG |
| tau3_R_F | TCGAAAATTCTGCGTTCGTTTTTTAGAATATATCTCTCCAATACAGCGTTAC |
| tau3_R_R | CGCGGATCCGCGGCCGCAAGTAATCGTCGAGACATTTATACAC |

**Supplementary Table S4** Plasmids used in this study.

| **Plasmids** | **Description** | **Source** |
| --- | --- | --- |
| pRS425K | *S. cerevisiae* multiple copy plasmid with *LEU2* and *KanMX* marker | This Lab |
| pGXJ01 | The cassette TDH2t-P_GAL1_-DHCR24-GGGGS-RFP-T_FBA1t_ was cloned and inserted into pRS425k | This study |
| pGXJ02 | The cassette TDH2t-P_GAL1_-AAMB-DHCR24-GGGGS-RFP-T_FBA1t_ was cloned and inserted into pRS425k | This study |
| pGXJ03 | The cassette TDH2t-P_GAL1_-AAMB-ERG2-GGGGS-RFP-T_FBA1t_ was cloned and inserted into pRS425k | This study |
| pGXJ04 | The cassette TDH2t-P_GAL1_-Oleosin-ERG3-GGGGS-RFP-T_FBA1t_ was cloned and inserted into pRS425k | This study |
| pGXJ05 | The cassette TDH2t-P_GAL1_-ERG7-GGGGS-GFP-T_FBA1t_ was cloned and inserted into pRS416 | This study |
| pGXJ06 | The cassette TDH2t-P_GAL1_-SEC61-GGGGS-GFP-T_FBA1t_ was cloned and inserted into pRS416 | This study |
| pGXJ07 | The cassette TDH2t-P_GAL1_-ERG3-T_FBA1t_ was cloned and inserted into pRS425k | This study |
| pGXJ08 | The cassette T_FBA1t_-P_GAL7_-ERG2-T_PGK1t_ was cloned and inserted into pRS425k | This study |
| pGXJ09 | The cassette TDH2t-P_GAL1_-Oleosin-ERG3-T_FBA1t_ was cloned and inserted into pRS425k | This study |
| pGXJ10 | The cassette T_FBA1t_-P_GAL7_-AAMB-ERG2-T_PGK1t_ was cloned and inserted into pRS425k | This study |
| pGXJ11 | The cassette TDH2t-P_GAL1_-ERG3-T_FBA1t_-P_GAL7_-ERG2-T_PGK1t_ was cloned and inserted into pRS425k | This study |
| pGXJ12 | The cassette TDH2t-P_GAL1_-Oleosin-ERG3-T_FBA1t_-P_GAL7_-AAMB-ERG2-T_PGK1t_ was cloned and inserted into pRS425k | This study |
| pGXJ13 | The cassette TDH2t-P_GAL1_-DHCR24-T_FBA1t_ was cloned and inserted into pRS425k | This study |
| pGXJ14 | The cassette TDH2t-P_GAL1_-AAMB-DHCR24-T_FBA1t_ was cloned and inserted into pRS425k | This study |
| pGXJ15 | Plasmid with gRNA of delta15 and cas9 | This study |
| pGXJ16 | The cassette delta15_L-TDH2t was cloned and inserted into pEAZY Blunt | This study |
| pGXJ17 | The cassette PGK1t-delta15_R was cloned and inserted into pEAZY Blunt | This study |
| pGXJ18 | The cassette HO_L-URA-TDH2t was cloned and inserted into pEAZY Blunt | This study |
| pGXJ19 | The cassette PGK1t-HO_R was cloned and inserted into pEAZY Blunt | This study |
| pGXJ20 | The cassette TDH2t-P_GAL1_-ERG25-GGGGS-ERG26-T_FBA1t_ was cloned and inserted into pRS425k | This study |
| pGXJ21 | The cassette T_FBA1t_-P_GAL7_-ERG27-T_PGK1t_-delta22_R was cloned and inserted into pRS425k | This study |
| pGXJ22 | The cassette TDH2t-P_GAL1_-ERG25-Oleosin-ERG26-T_FBA1t_ was cloned and inserted into pRS425k | This study |
| pGXJ23 | The cassette T_FBA1t_-P_GAL7_-AAMB-ERG27-T_PGK1t_-delta22_R was cloned and inserted into pRS425k | This study |
| pGXJ24 | The cassette TDH2t-P_GAL1_-ERG1-GGGGS-ERG11-T_FBA1t_ was cloned and inserted into pRS425k | This study |
| pGXJ25 | The cassette T_FBA1t_-P_GAL7_- ERG24-T_PGK1t_-delta22_R was cloned and inserted into pRS425k | This study |
| pGXJ26 | The cassette TDH2t-P_GAL1_-ERG1-Oleosin-ERG11-T_FBA1t_ was cloned and inserted into pRS425k | This study |
| pGXJ27 | The cassette T_FBA1t_-P_GAL7_- AAMB-ERG24-T_PGK1t_-delta22_R was cloned and inserted into pRS425k | This study |
| pGXJ28 | The cassette T_FBA1t_-P_GAL7_- ERG24-T_PGK1t_-delta15_R was cloned and inserted into pRS425k | This study |
| pGXJ29 | The cassette T_FBA1t_-P_GAL7_- AAMB-ERG24-T_PGK1t_-delta15_R was cloned and inserted into pRS425k | This study |
| pGXJ30 | The cassette delta22_L-TDH2t was cloned and inserted into pEAZY Blunt | This study |
| pGXJ31 | Plasmid with gRNA of delta22 and cas9 | This study |
